# There Is Significant Within-Subject Variation in the Time from Light Stimulus to Maximum Pupil Constriction Among Healthy Controls

**DOI:** 10.3390/jcm13237451

**Published:** 2024-12-06

**Authors:** Abdulkadir Kamal, Yohan Kim, Amber Salter, Shripal Gunna, Emerson B. Nairon, DaiWai M. Olson

**Affiliations:** Department of Neurology, The University of Texas Southwestern Medical Center (UTSW), Dallas, TX 75390, USA; kamal.abdulkadir@utsouthwestern.edu (A.K.); amber.salter@utsouthwestern.edu (A.S.); shripal.gunna@utsouthwestern.edu (S.G.); emerson.nairon@utsouthwestern.edu (E.B.N.)

**Keywords:** pupil light reflex, quantitative pupillometry, pupils, autonomic nervous system, neurocritical care, assessment

## Abstract

**Background**: Handheld quantitative pupilometers (QPs) measure each phase of the pupillary light reflex (PLR) and provide a summary score based on these values. One phase of the PLR is the period of time from the onset of light exposure to the maximum constriction of the pupil, also known as time to maximum constriction (tMC). Although tMC has been found to vary significantly among patients with neurological injury, there are no studies reporting tMC in healthy controls. This study addresses this gap. **Methods**: Subjects in this prospective observational study were healthy controls who provided paired (left and right eye) QP readings during four separate observations over the course of 2 days. The tMC was derived by determining the smallest observed pupil size during videos filmed at 30 frames per second, and we assessed within-subject variability using the coefficient of variance and intraclass correlation coefficient (ICC). **Results**: Fifty subjects provided 380 QP readings (190 left eye and 190 right eye). Subjects primarily identified as female (80%), non-Hispanic (86%), white (62%), and <40 years old (74%). The mean tMC was 1.0 (0.14) seconds (s) for the left eye and 1.0 (0.17) s for the right eye; the coefficient of variance ranged from 11.6% to 18.8% and the ICC ranged from 0.25 to 0.40. For the between-subject comparisons across the four observation periods, the left and right eye mean differences ranged from 0.001 to 0.063 and the ICC ranged from 0.12 to 0.52. **Conclusions**: The tMC values vary significantly in healthy controls. Changes in pupil function as a clinical biomarker of intracranial pathology are not fully understood. Identifying clinical correlations of tMC variation may provide insight for the prognostication and treatment of neurocritically ill patients.

## 1. Introduction

The pupillary light reflex (PLR) exam is standard-of-care in the neuroscience intensive care unit (NSICU) for all patients and documented on an hourly basis [1,2]. The PLR is the complex pathway controlled by the brain that involves both the sympathetic (pupil dilation) and parasympathetic (pupil constriction) responses to light [3]. This pathway has been characterized as having relatively consistent timepoints with constriction occurring at about 1 s and the dilation phase lasting an additional 5 s [4]. The afferent pathway of the PLR begins when light falls on the retina and a signal is transmitted through the optic nerve, chiasm, and optic tract. The signal synapses at the pretectal olivary nucleus then project to both the ipsilateral and contralateral Edinger–Westphal nuclei. Parasympathetic efferent signals are then transmitted through the oculomotor nerve, synapsing with nuclei in the ciliary ganglion and traveling to the sphincter pupillae, resulting in pupillary constriction [5]. Improving our understanding of the various mechanisms that control the amount of light that enters the eye and how certain neurological illnesses or injuries can interfere with this process will improve diagnosis, prognosis, and treatment [6,7].

While pupil reactivity assessment has been integral to critical care for many years, it has inadequate interrater reliability to provide consistent information that can be trended to reflect change over time [8]. Recent advances in technology have changed practice [9], and the use of quantitative pupillometer (QP) devices is becoming increasingly common in clinical practice [10]. These advances have allowed the PLR exam to evolve from a subjective assessment without rigid guidelines to a reliably objective measurement that provides accurate diagnostic information for clinicians [11,12]. Data from QP demonstrating that a sluggish pupil can be normally reactive and a brisk pupil can be abnormal provide additional evidence that even if subjective assessments were reliable, they are insufficiently precise [13,14]. As a result, there is a better understanding of the overall function of the pupil.

QP has high reliability and is being increasingly adopted in clinical practice to replace the subjectivity and low reliability associated with clinical assessments [15,16]. By breaking down the PLR into discrete components, QP provides a variety of measures representing changes in pupil diameter [17]. Pupil size in diameter is reported first as the size prior to light stimulus (size) and again as the smallest observed diameter (maximum constriction) of the pupil [18]. Latency is measured in fractions of a second and represents the time from light stimulus until the onset of constriction [19]. Constriction velocity (CV) is reported as millimeters (mm) per second (s) and reflects the change in pupil size from the end of latency until maximum constriction [13,20]. Dilation velocity is measured in mm/s and reflects the speed of pupil dilation after maximum constriction [21]. The neurological pupil index (NPi) is a scalar value summary score that quantifies the PLR; this value ranges from 0 to 5 [22]. An NPi of 3.0 or higher is considered a normal pupillary reflex; an NPi value below 3.0 is indicative of an abnormal pupillary response [23,24]. An NPi < 3.0 suggests a variety of intracranial pathologies including increased intracranial pressure or changes in sympathetic and parasympathetic pathways [6,9]. In addition to influence from autonomic nervous system (ANS) changes, changes in PLR can indicate intracranial hemorrhage, mass effect, or an inflammatory condition [7,25,26].

**Figure 1:**
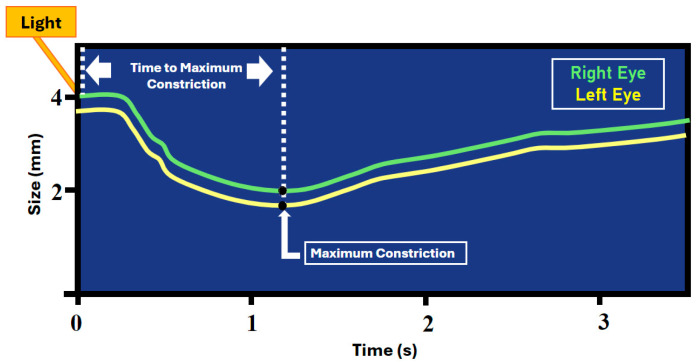
Highlighting the time to maximum constriction.

Though the discrete components of the PLR are increasingly reported in the recent literature, there is a gap in research regarding the time to maximum constriction (tMC). As shown in Figure 1, tMC is the time from light exposure to the smallest identified pupil size [19]. Only recently reported in the literature, the tMC of patients with acquired brain injury was noted to have significant between-subject variation [19]. Before the relevance of tMC as a novel biomarker can be fully examined, there is a need to identify the normal tMC values in the absence of cerebral injury. Defining normative tMC values will facilitate discovery of the causes for variance in PLR response time from pupillary light exposure to the point of maximum pupil constriction that have not been defined. This observational study aims to determine if a consistent tMC can be identified in a cohort of healthy subjects. The null hypothesis is that there is no significant within-subject variance in tMC across repeated observations.

## 2. Materials and Methods

This prospective non-randomized observational study enrolled 50 healthy subjects working in the NSICU at a university hospital in the Southwest. The study received institutional review board (IRB) approval to enroll university hospital employees. All full- and part-time employees working in the NSICU were eligible, but medical residents and students were excluded as the IRB deemed them a vulnerable population. Eligible subjects met the inclusion criteria if they were >18 years old, had no history of eye injury, had bilateral reactive pupils, and were expected to be available 1 h after their first QP reading was obtained and again 24 h after the second QP reading was obtained. Subjects with any medical consideration of the eye (e.g., glaucoma, cataract, conjunctivitis, prothesis, etc.) were considered ineligible. Written consent was waived by the IRB, and eligible employees were verbally consented during both the day shift (7 a.m.–7 p.m.) and the night shift (7 a.m.–7 p.m.). To protect subject anonymity, age was trichotomized as <40 years, 41–59 years, or ≥60 years. To reflect real-world conditions, the QP readings were obtained at a time and location convenient to the subject without changing the current ambient light level, and no limits were placed on the healthy subjects regarding caffeine intake or requirement of physical activity or sleep.

**Figure 2:**
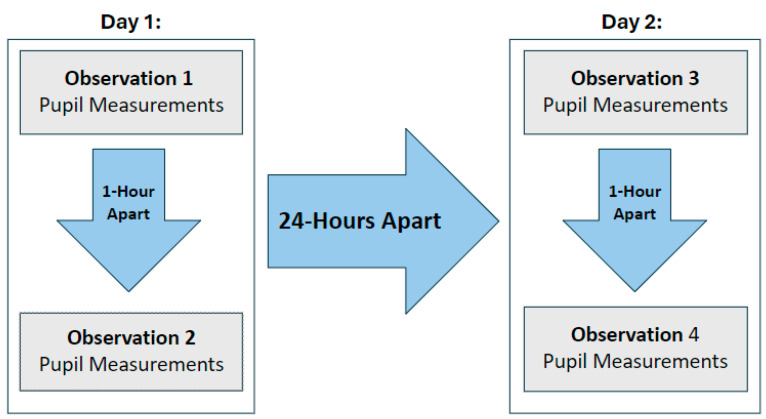
Data collection plan for QP readings during the 4 observation periods.

All data collection occurred within the NSICU but in varying locations, times, and ambient light levels. QP data were obtained using the NPi-300 device (NeurOptics Inc., Irvine, CA, USA) which, by default, filters out all light except infrared. All samples were collected by research personnel trained in the use of the QP device. To facilitate data collection without impacting the volunteers’ ability to provide direct patient care, no restrictions were placed on the time of day at which the 1st observation was obtained. The QP readings were obtained with the subject standing and gazing horizontally and the device held perpendicular to the eye. Subjects were instructed to look straight ahead and focus on an object over the shoulder of the person obtaining the QP reading. Subjects were asked not to blink; upon consent, the examiner gently held up the eyelid for subjects with an exaggerated blink reflex. Every volunteer subject provided 8 separate QP readings, 1 from each eye during 4 observations. The 1st and 2nd observations were 1 h apart on day 1. The 3rd and 4th observations were 1 h apart on day 2. The 2nd and 3rd observations were at least 24 h apart (Figure 2). All data were obtained between 13 August and 28 August 2024.

After each QP reading, the data were uploaded from a microchip on the disposable eyepiece (SmartGuard^®^, Neuroptics, Irvine, CA, USA) to an electronic spreadsheet (Excel^TM^, Microsoft Inc, Redmond, WA, USA). The device filmed images at 30 frames per second and the time to maximum constriction was derived as 0.033 times the number of frames from onset of light stimulus to the smallest recorded pupil size and recorded as thousandths of a second. Demographic data were linked to QP data using a de-identified subject number.

The sample size was determined using data from a similar study design conducted with a sample of critically ill patients. Assuming that at least a 10% difference in variance in tMC is required to reject the null hypothesis, a minimum of 21 independent observations are required (1 − β = 0.80, α = 0.05). The mean difference (∆), coefficient of variation (CoVar), and intraclass correlation coefficient (ICC) were calculated for within-subject and between-observation comparisons. Within-subject comparisons examined paired (left eye versus right eye) comparisons at each of the 4 observation periods. Between-observation comparisons were explored by creating models to explore the consistency of all left-eye tMC values for all combinations of the 4 observation periods separately and repeating those comparisons for the right-eye tMC values. Data were analyzed using SAS v9.4 for Windows (SAS Institute, Cary, NC, USA).

## 3. Results

There were 190 paired PLR readings collected from 50 healthy subjects. Ten readings were lost to follow-up from five subjects that were not available for subsequent scanning. As shown in Table 1, 37 (74%) subjects were less than 40 years old, 40 (80%) were female, and 43 (86%) identified as non-Hispanic. There were 10 (20%) subjects who identified as black, 31 (62%) as white, and 8 (16%) as Asian; 1 (2%) subject did not categorize their race or ethnicity.

The tMC values for the left and right eye were first examined independently. Each separate QP metric value is reported in Table 2 and these were found to be approximately normally distributed. The left eye mean tMC was 1.0 (0.14) s and the right eye mean tMC was 1.0 (0.17) s.

Table 3 provides results for within-subject comparisons of the left versus right eye tMC values in each of the four observation periods. The mean tMC values range from 0.995 to 1.050 s and the coefficient of variation (CoVar) values range from 11.6% to 18.8%. The mean difference (∆) ranged from 0.12 to 0.14 and the ICC ranged from 0.25 to 0.40.

**Figure 3:**
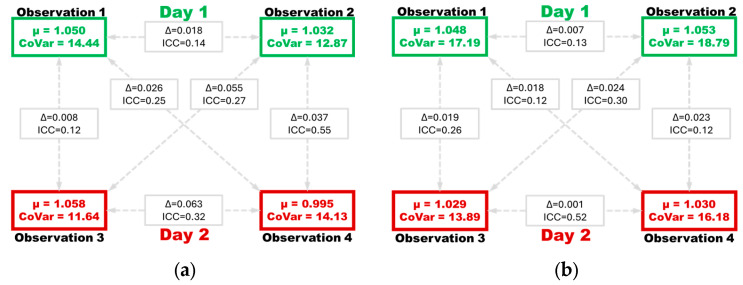
Showing the mean (µ) coefficient of variation (CoVar) time to maximum constriction (tMC) values during each observation period, as well as the mean difference (∆) and intraclass correlation coefficient (ICC) values of each comparison, showing left eye values (**a**) and right eye values (**b**) separately.

As shown in Figure 3, the between-observation comparisons were performed to evaluate differences in left-eye tMC values between all four observation periods (Figure 3a) separately from all right-eye tMC values (Figure 3b). The ∆ ranged from 0.008 to 0.063 s for the left eye and ranged from 0.001 to 0.024 s for the right eye. The ICC values ranged from 0.12 to 0.55 for the left eye and ranged from 0.12 to 0.52 for the right eye.

## 4. Discussion

Within-subject tMC values are significantly different over time. These data support the conclusion that there is significant within-eye and between-observation variation in tMC in healthy subjects under ambient light conditions. Additionally, there is poor reliability of the tMC values between eyes and observations. These data extend results from a study examining tMC among patients with neurological injury which showed that tMC varied by up to 0.89 s between patients and 0.56 s within patients [19]. Given that there is a variance in tMC that is not fully explained by pupil size, latency, and constriction velocity, we must conclude that there are other factors which may be contributing to this variance. The finding that tMC variance is similar in both healthy subjects without pathology and patients with abnormal neurological pathology suggests that the tMC is determined by the ANS.

Identifying a means to rule out, or rule in, ANS dysregulation as a causative agent for an abnormal PLR could benefit prognostication and treatment of patients with acquired brain injury [27]. The timing of interventions has been linked to improvement in outcome for patients at risk for secondary brain injury [28,29]. For patients who arrive with a disorder of consciousness, it is imperative to identify the cause, yet computerized tomography (CT) of the brain can look normal shortly after injury that may evolve [30]. In a patient without brain injury, the ability to identify ANS dysregulation could similarly have a clinical benefit. Testing the ANS typically includes a collection of tests because no single test has adequately high sensitivity and specificity as of yet [31]. If the tMC can be linked to ANS, it may, in a multivariable model, improve ANS testing.

Understanding tMC will also provide clinical relevance for a host of novel PLR measurements. A recently identified clinical variable is anisocoria after light emission [32]. In contrast to anisocoria observed prior to light stimulus, anisocoria after light is not dependent on baseline pupil size or variables that impact size (e.g., caffeine). Moreover, anisocoria after light is a better predictor of outcome after brain injury than anisocoria at baseline [33]. Because anisocoria after light is measured as the maximum constriction, there is a need to further identify factors such as tMC that impact maximum constriction.

As of yet, we are unable to isolate biomarkers along the PLR pathway responsible for tMC variance. Although the overall mean tMC was 1.0 s and roughly 178/390 (45.6%) tMC values were between 0.9 s and 1.1 s, the range of observed values from 0.67 s to 1.80 s warrants further investigation. Assuming that ANS plays a role in tMC, changes in either afferent or efferent pathways may influence tMC variance [34].

The CoVar values ranged from 11.6% to 18.8%, indicating moderate dispersion of the within-subject tMC values. The ICC values ranged from 0.25 to 0.40, indicating poor to fair agreement between paired tMC readings. This indicates that initial tMC values have low reliability and considerable variability in tMC values over short intervals, as shown in Figure 3. As discussed in similar studies, PLR metrics are potentially influenced by various factors, such as ambient light levels at the time of each observation [35]. Extrinsic factors may explain the moderate variation in tMC values as well as the poor to fair pairing of tMC values across successive scans, as various factors can influence the PLR. However, the study design removes the potential influence of intrinsic factors.

It remains possible that tMC variance is associated with identifiable uncontrolled confounders in our study, such as medication use, caffeine, or sleep hygiene. Specifically, circadian rhythm has been identified to impact PLR function [36]. Although samples were obtained at pragmatic intervals, they were ordinal and 1 h apart. Given that there was no identifiable direction between the first and second observation on each day (Figure 3), we suggest that circadian rhythm does not influence tMC variation. While evidence suggests that caffeine impacts pupil diameter [37], a meta-analysis in 2024 [38] found that there is, as of yet, insufficient evidence that caffeine impacts PLR. The impact of caffeine or other medications (especially those hypothesized to impact the autonomic nervous system) remains worthy of study, but these variables were not recorded in the present study.

### Limitations

Ambient light levels were not controlled during measurement; however, this replicates real-world scenarios in which pupillometry would be used by nurses and nursing assistants [39]. Sample sizes and collection of demographic data limit the ability to consider the effect of factors such as age, sex, and race on the reliability and variability and limit generalizability. Future research standardizing ambient light exposure to observe how this can influence the tMC is warranted. All measurements of PLR were performed during the time when subjects were available at work and the study team were also at work. Measurements were not taken during the evening time or early morning time, during which subjects would be in different emotional and physical states that would perhaps affect the PLR exam. 

## 5. Conclusions

The findings in this study provide data that are sufficient to reject the null hypothesis and conclude that tMC is variable in healthy subjects and it cannot be predetermined as a set value on a group or individual level. Non-critically ill subjects experience tMC variance, and this variance is found both within eyes and over short (hours) and long (days) intervals of time. Pupil constriction, as a clinical biomarker, is well studied but not well understood. The more we understand the relationship between pupil constriction and varying intracranial pathology, the better we can prognosticate and treat neurocritically ill patients. The findings in this observational study broaden the understanding of the metrics of QP tMC and related pathologic neurological phenomena.

## Figures and Tables

**Table 1:** Subject characteristics.

Characteristic		Subjects
Age	<40 Years 40–59 Years ≥60 years	37 (74.0%) 9 (18.0%) 4 (8.0%)
Gender	Female Male	40 (80.0%) 10 (20.0%)
Race	White Black Asian Not given	31 (62.0%) 10 (20.0%) 8 (16.0%) 1 (2.0%)
Ethnicity	Non-Hispanic Hispanic Not given	43 (86.0%) 6 (12.0%) 1 (2.0%)

**Table 2:** Discrete values for each of the pupillary light reflex metrics among the 380 pupillometer readings.

Side	Pupillary Light Reflex Metric	Mean (sd)	Units
Left Eye (*n* = 190)	Neurological pupil index	4.3 (0.33)	
	Diameter of pupil before light stimulation	4.2 (0.81)	mm
	Time between stimulus and initial constriction (latency)	0.2 (0.03)	s
	Constriction velocity (CV)	2.6 (0.82)	mm/s^2^
	Maximum constriction velocity (mCV)	3.8 (1.14)	mm/s^2^
	Smallest diameter of pupil after light stimulation	2.8 (0.45)	mm
	Time from stimulus to maximum constriction (tMC)	1.0 (0.14)	s
	Percent change in pupil size after light (DV)	31.9 (6.61)	%
	Dilation velocity (DV)	1.1 (0.31)	mm/s^2^
Right Eye (*n* = 190)	Neurological pupil index	4.3 (0.31)	
	Diameter of pupil before light stimulation	4.2 (0.80)	mm
	Time between stimulus and initial constriction (latency)	0.2 (0.03)	s
	Constriction velocity (CV)	2.6 (0.77)	mm/s^2^
	Maximum constriction velocity (mCV)	3.8 (1.12)	mm/s^2^
	Smallest diameter of pupil after light stimulation	2.8 (0.43)	mm
	Time from stimulus to maximum constriction (tMC)	1.0 (0.17)	s
	Percent change in pupil size after light	32.0 (6.7)	%
	Dilation velocity (DV)	1.1 (0.31)	mm/s^2^

**Table 3:** Mean difference (∆) coefficient of variation (CoVar) and interclass correlation coefficient (ICC) for the left and right eye time to maximum constriction (tMC).

Day	Observation Period	Left Eye tMC Mean (CoVar)	Right Eye tMC Mean (CoVar)	∆ (sd)	ICC (95%CI)
1	1	1.050 (14.44)	1.048 (17.19)	0.14 (0.15)	0.25 (0.08–0.57)
	2	1.032 (12.87)	1.032 (18.79)	0.14 (0.12)	0.38 (0.19–0.63)
2	3	1.058 (11.64)	1.058 (13.89)	0.12 (0.09)	0.40 (0.20–0.65)
	4	0.995 (14.13)	0.995 (16.18)	0.12 (0.12)	0.35 (0.15–0.62)

## Data Availability

The data that support the findings of this study are available upon reasonable request.

## References

[1] Brazel M., Harris J., Carroll D., Davidson J., Levchak P.J., Malhotra A., LaBuzetta J.N. (2024). Reporting on Neurological Decline as Identified by Hourly Neuroassessments. J. Neurosci. Nurs..

[2] Banzon P.C., Vashisht A., Euckert M., Nairon E., Aiyagari V., Stutzman S.E., Olson D.M. (2023). Original Research: Practice Variations in Documenting Neurologic Examinations in Non-Neuroscience ICUs. Am. J. Nurs..

[3] Lapierre A., Proulx A., Gélinas C., Dollé S., Alexander S., Williamson D., Bernard F., Arbour C. (2024). Association Between Pupil Light Reflex and Delirium in Adults With Traumatic Brain Injury: Preliminary Findings. J. Neurosci. Nurs..

[4] Ciuffreda K.J., Joshi N.R., Truong J.Q. (2017). Understanding the effects of mild traumatic brain injury on the pupillary light reflex. Concussion.

[5] Gupta A., Bansal R., Sharma A., Kapil A., Gupta A., Bansal R., Sharma A., Kapil A. (2023). Pupillary Signs. Ophthalmic Signs in Practice of Medicine.

[6] Martinez-Palacios K., Vasquez-Garcia S., Fariyike O.A., Robba C., Rubiano A.M., Noninvasive Intracranial Pressure Monitoring International Consensus Group (2024). Quantitative Pupillometry for Intracranial Pressure (ICP) Monitoring in Traumatic Brain Injury: A Scoping Review. Neurocrit. Care.

[7] Aoun S.G., Welch B.G., Cortes M., Stutzman S.E., MacAllister M.C., El Ahmadieh T.Y., Osman M., Figueroa S.A., White J.A., Batjer H.H. (2019). Objective Pupillometry as an Adjunct to Prediction and Assessment for Oculomotor Nerve Injury and Recovery: Potential for Practical Applications. World Neurosurg..

[8] Olson D.M., Stutzman S., Saju C., Wilson M., Zhao W., Aiyagari V. (2016). Interrater Reliability of Pupillary Assessments. Neurocrit. Care.

[9] Figueroa S.A., Olson D.M., Kamal A., Aiyagari V. (2024). Quantitative Pupillometry: Clinical Applications for the Internist. Am. J. Med..

[10] Lele A.V., Wahlster S., Khadka S., Walters A.M., Fong C.T., Blissitt P.A., Livesay S.L., Jannotta G.E., Gulek B.G., Srinivasan V. (2023). Neurological Pupillary Index and Disposition at Hospital Discharge following ICU Admission for Acute Brain Injury. J. Clin. Med..

[11] Zheng D., Huang Z., Chen W., Zhang Q., Shi Y., Chen J., Cen L., Li T. (2022). Repeatability and clinical use of pupillary light reflex measurement using RAPDx(R) pupillometer. Int. Ophthalmol..

[12] Romagnoli S., Lobo F.A., Picetti E., Rasulo F.A., Robba C., Matta B. (2024). Non-invasive technology for brain monitoring: Definition and meaning of the principal parameters for the International PRactice On TEChnology neuro-moniToring group (I-PROTECT). J. Clin. Monit. Comput..

[13] Shoyombo I., Aiyagari V., Stutzman S.E., Atem F., Hill M., Figueroa S.A., Miller C., Howard A., Olson D.M. (2018). Understanding the Relationship Between the Neurologic Pupil Index and Constriction Velocity Values. Sci. Rep..

[14] Privitera C.M., Neerukonda S.V., Aiyagari V., Yokobori S., Puccio A.M., Schneider N.J., Stutzman S.E., Olson D.M. (2022). A differential of the left eye and right eye neurological pupil index is associated with discharge modified Rankin scores in neurologically injured patients. BMC Neurol..

[15] Smith J., Flower O., Tracey A., Johnson P. (2020). A comparison of manual pupil examination versus an automated pupillometer in a specialised neurosciences intensive care unit. Aust. Crit. Care.

[16] Blandino Ortiz A., Higuera Lucas J. (2022). Usefulness of quantitative pupillometry in the intensive care unit. Med. Intensiv. (Engl. Ed.).

[17] Olson D.M., Fishel M. (2016). The Use of Automated Pupillometry in Critical Care. Crit. Care Nurs. Clin. N. Am..

[18] Lussier B.L., Stutzman S.E., Atem F., Venkatachalam A.M., Perera A.C., Barnes A., Aiyagari V., Olson D.M. (2019). Distributions and Reference Ranges for Automated Pupillometer Values in Neurocritical Care Patients. J. Neurosci. Nurs..

[19] Kamal A., Nairon E.B., Bashmakov A., Aoun S.G., Olson D.M. (2024). Time to maximum pupil constriction is variable in neurocritical care patients. J. Clin. Monit. Comput..

[20] Shao L., Zhou Y., Yue Z., Gu Z., Zhang J., Hui K., Xiong J., Xu M., Duan M. (2022). Pupil maximum constriction velocity predicts post-induction hypotension in patients with lower ASA status: A prospective observational study. BMC Anesthesiol..

[21] Uhrenholt S., Linér S.M., Stokholm J., Christensen T., Bestle M.H. (2024). Pupillary dilation velocity is reduced in intensive care unit patients with septic shock. Acta Anaesthesiol. Scand..

[22] Romagnosi F., Bernini A., Bongiovanni F., Iaquaniello C., Miroz J.P., Citerio G., Taccone F.S., Oddo M. (2022). Neurological Pupil Index for the Early Prediction of Outcome in Severe Acute Brain Injury Patients. Brain Sci..

[23] Lussier B.L., Olson D.M., Aiyagari V. (2019). Automated Pupillometry in Neurocritical Care: Research and Practice. Curr. Neurol. Neurosci. Rep..

[24] Jiang J., Sari H., Goldman R., Huff E., Hanna A., Samraj R., Gourabathini H., Bhalala U. (2023). Neurological Pupillary Index (NPi) Measurement Using Pupillometry and Outcomes in Critically Ill Children. Cureus.

[25] Campos Y.A., Rana P., Reyes R.G., Mazhar K., Stutzman S.E., Atem F., Olson D.M., Aiyagari V. (2022). Relationship Between Automated Pupillometry Measurements and Ventricular Volume in Patients With Aneurysmal Subarachnoid Hemorrhage. J. Neurosci. Nurs..

[26] Brasil S., Frigieri G., Taccone F.S., Robba C., Solla D.J.F., de Carvalho Nogueira R., Yoshikawa M.H., Teixeira M.J., Malbouisson L.M.S., Paiva W.S. (2023). Noninvasive intracranial pressure waveforms for estimation of intracranial hypertension and outcome prediction in acute brain-injured patients. J. Clin. Monit. Comput..

[27] Murtaugh B., Olson D.M., Badjatia N., Lewis A., Aiyagari V., Sharma K., Creutzfeldt C.J., Falcone G.J., Shapiro-Rosenbaum A., Zink E.K. (2024). Caring for Coma after Severe Brain Injury: Clinical Practices and Challenges to Improve Outcomes: An Initiative by the Curing Coma Campaign. Neurocrit. Care.

[28] Chau C.Y.C., Mediratta S., McKie M.A., Gregson B., Tulu S., Ercole A., Solla D.J.F., Paiva W.S., Hutchinson P.J., Kolias A.G. (2020). Optimal Timing of External Ventricular Drainage after Severe Traumatic Brain Injury: A Systematic Review. J. Clin. Med..

[29] Olson D.M., Ortega-Pérez S. (2019). The Cue-Response Theory and Nursing Care of the Patient With Acquired Brain Injury. J. Neurosci. Nurs..

[30] Chairattanawan P., Angkoontassaneeyarat C., Yuksen C., Jenpanitpong C., Phontabtim M., Laksanamapune T. (2024). Early Discharge versus 6-hour Observation in Mild Traumatic Brain Injury with Normal Brain CT Scan; a Comparative Pilot study of Outcomes. Arch. Acad. Emerg. Med..

[31] Baker J.R., Hira R., Uppal J., Raj S.R. (2024). Clinical Assessment of the Autonomic Nervous System. Card. Electrophysiol. Clin..

[32] Saju C., Barnes A., Kuramatsu J.B., Marshall J.L., Obinata H., Puccio A.M., Yokobori S., Olson D.M. (2023). Describing Anisocoria in Neurocritically Ill Patients. Am. J. Crit. Care.

[33] Doyle B.R., Aiyagari V., Yokobori S., Kuramatsu J.B., Barnes A., Puccio A., Nairon E.B., Marshall J.L., Olson D.M. (2024). Anisocoria After Direct Light Stimulus is Associated with Poor Outcomes Following Acute Brain Injury. Neurocrit. Care.

[34] Vrettou C.S., Fragkou P.C., Mallios I., Barba C., Giannopoulos C., Gavrielatou E., Dimopoulou I. (2024). The Role of Automated Infrared Pupillometry in Traumatic Brain Injury: A Narrative Review. J. Clin. Med..

[35] Aderman M.J., Meister M.R., Roach M.H., Dengler B.A., Ross J.D., Malvasi S.R., Cameron K.L. (2024). Normative Values for Pupillary Light Reflex Metrics Among Healthy Service Academy Cadets. Mil. Med..

[36] Bonmati-Carrion M.A., Hild K., Isherwood C., Sweeney S.J., Revell V.L., Skene D.J., Rol M.A., Madrid J.A. (2016). Relationship between Human Pupillary Light Reflex and Circadian System Status. PLoS ONE.

[37] Abokyi S., Owusu-Mensah J., Osei K.A. (2017). Caffeine intake is associated with pupil dilation and enhanced accommodation. Eye.

[38] Hartmann E.V., Reichert C.F., Spitschan M. (2024). Effects of caffeine intake on pupillary parameters in humans: A systematic review and meta-analysis. Behav. Brain Funct..

[39] Stout D.E., Cortes M.X., Aiyagari V., Olson D.M. (2019). Management of External Ventricular Drains During Intrahospital Transport for Radiographic Imaging. J. Radiol. Nurs..

